# High Levels of Detection of Nonpneumococcal Species of Streptococcus in Saliva from Adults in the United States

**DOI:** 10.1128/spectrum.05207-22

**Published:** 2023-04-17

**Authors:** Maikel S. Hislop, Orchid M. Allicock, Darani A. Thammavongsa, Sidiya Mbodj, Allison Nelson, Albert C. Shaw, Daniel M. Weinberger, Anne L. Wyllie

**Affiliations:** a Department of Epidemiology of Microbial Diseases, Yale School of Public Health, New Haven, Connecticut, USA; b Department of Internal Medicine, Section of Infectious Diseases, Yale School of Medicine, New Haven, Connecticut, USA; Memorial Sloan Kettering Cancer Center

**Keywords:** saliva, pneumococcus, molecular diagnostics, qPCR, streptococci

## Abstract

While the sensitivity of detection of pneumococcal carriage can be improved by testing respiratory tract samples with quantitative PCR (qPCR), concerns have been raised regarding the specificity of this approach. We therefore investigated the reliability of the widely used *lytA* qPCR assay when applied to saliva samples from older adults in relation to a more specific qPCR assay (*piaB*). During the autumn/winter seasons of 2018/2019 and 2019/2020, saliva was collected at multiple time points from 103 healthy adults aged 21 to 39 (*n* = 34) and >64 (*n* = 69) years (*n* = 344 total samples). Following culture enrichment, extracted DNA was tested using qPCR for *piaB* and *lytA*. By sequencing the variable region of *rpsB* (S2 typing), we identified the species of bacteria isolated from samples testing *lytA*-positive only. While 30 of 344 (8.7%) saliva samples (16.5% individuals) tested qPCR-positive for both *piaB* and *lytA*, 52 (15.1%) samples tested *lytA*-positive only. No samples tested *piaB*-positive only. Through extensive reculture attempts of the *lytA*-positive samples collected in 2018/2019, we isolated 23 strains (in 8 samples from 5 individuals) that were also qPCR-positive for only *lytA*. Sequencing determined that Streptococcus mitis and Streptococcus infantis were predominantly responsible for this *lytA*-positive qPCR signal. We identified a comparatively large proportion of samples generating positive signals with the widely used *lytA* qPCR and identified nonpneumococcal Streptococcus species responsible for this signal. This highlights the importance of testing for the presence of multiple gene targets in tandem for reliable and specific detection of pneumococcus in polymicrobial respiratory tract samples.

**IMPORTANCE** Testing saliva samples with quantitative PCR (qPCR) improves the sensitivity of detection of pneumococcal carriage. The qPCR assay targeting *lytA*, the gene encoding the major pneumococcal autolysin, has become widely accepted for the identification of pneumococcus and is even considered the “gold standard” by many. However, when applying this approach to investigate the prevalence of pneumococcal carriage in adults in New Haven, CT, USA, we identified nonpneumococcal Streptococcus spp. that generate positive signals in this widely used assay. By testing also for *piaB* (encoding the iron acquisition ABC transporter lipoprotein, PiaB), our findings demonstrate the importance of testing for the presence of multiple gene targets in tandem for reliable molecular detection of pneumococcus in respiratory tract samples; targeting only *lytA* may lead to an overestimation of true carriage rates.

## INTRODUCTION

Upper respiratory tract carriage of Streptococcus pneumoniae (pneumococcus) is considered a prerequisite for invasive pneumococcal disease (IPD). Rates of carriage are highest in young children (60% to 80%) (1–3). Carriage of pneumococcus is less commonly detected in older adults through a culture-based approach (≤5%) (4–8), despite a high incidence of disease in this age group (9). Recent studies have demonstrated that when more sensitive methods are used, namely, quantitative PCR (qPCR) (10–12), higher rates of carriage in this at-risk age group can be detected, especially when using samples from oral sites (4, 13, 14).

For the detection of pneumococcus by qPCR, a suitable gene target must be selected to ensure both sensitive and specific identification; multiple gene targets further increase specificity, reducing false-positive detection of pneumococcus (15). However, the majority of molecular assays available for the detection of pneumococcus are developed using pure isolates, isolates in cell culture, or isolates from nasopharyngeal swabs. Thus, these assays do not account for the complicated microbial composition of the oropharynx/oral cavity and the potential for detection of closely related nonpneumococcal Streptococcus spp. (16–18).

The variability in sample types tested and detection methods applied (culture versus molecular) leads to difficulty comparing results between settings. In the current study, we validated and evaluated previously reported methods (4, 19) in which culture-enriched saliva samples are tested in qPCR for the pneumococcus-specific genes *lytA* (considered by many as the gold standard for qPCR detection of pneumococcus) (13) and *piaB* (increasingly being evaluated alongside *lytA* to improve the specificity of detection of pneumococcus) (3, 11). The frequent identification of nonpneumococcal Streptococcus spp. in individuals testing positive for *lytA* alone highlights the importance of testing multiple gene targets (both *lytA* and *piaB*) to obtain reliable detection.

## RESULTS

### Population characteristics.

**FIG 1:**
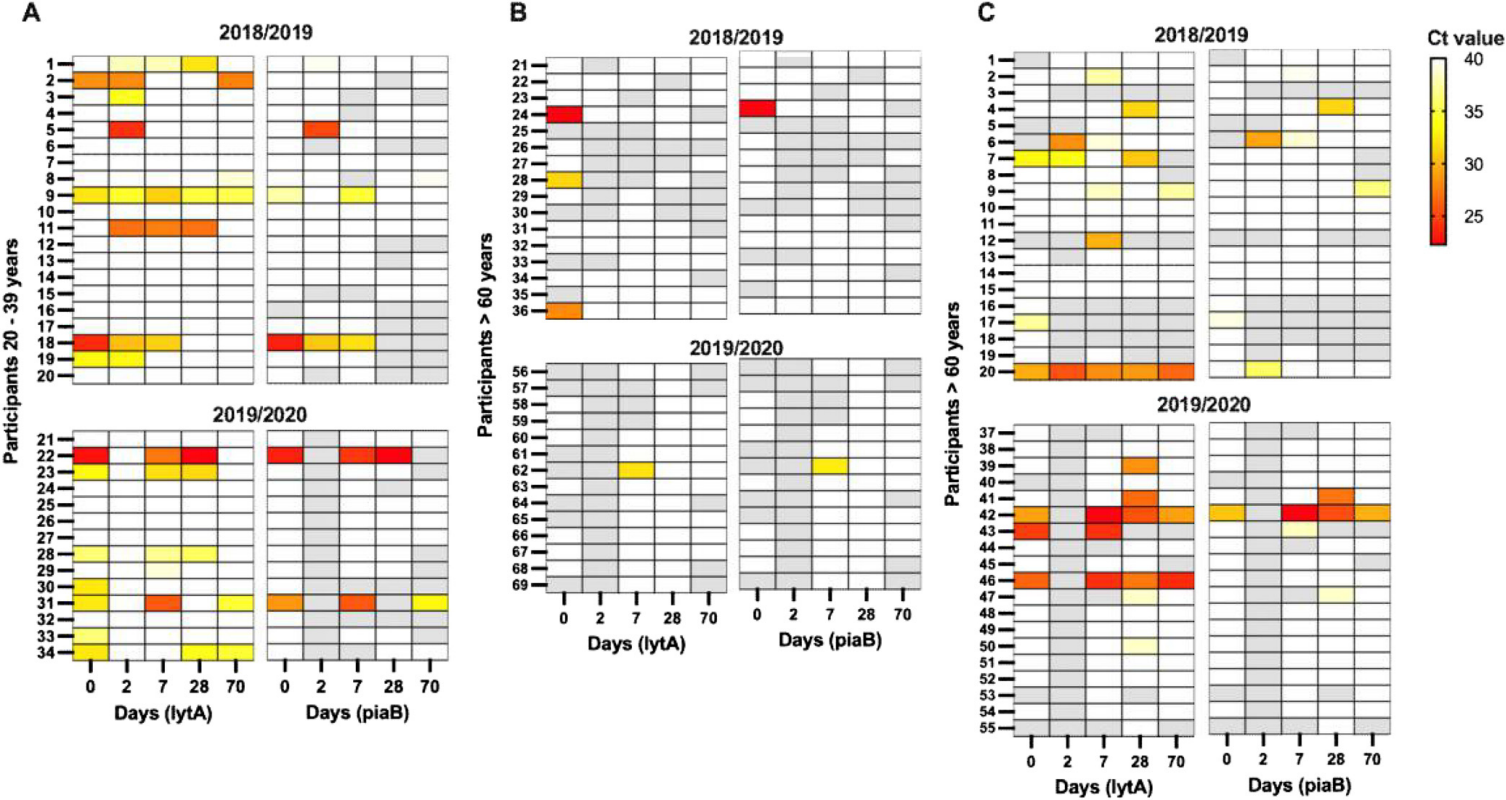
Detection of pneumococcal genes *lytA* and *piaB* in saliva samples from healthy adults aged 21 to 39 years (A) and 60 years and older (B, C) residing in an aged-care living facility (B) or in the community (C), collected during the autumn/winter seasons of 2018/2019 and 2019/2020. Each row represents a study participant, and each column is a sample collected on the indicated day, following influenza vaccination. Samples represented by darker colors indicate a higher density of bacteria (lower PCR Ct value), lighter colors indicate a lower density of bacteria (higher PCR Ct value), white indicates no detection of the gene target, and gray indicates the sample was not available for testing (either not collected or insufficient volume).

A total of 344 samples was collected from 103 individuals over the course of the two study seasons (Fig. 1). During the 2018/2019 study period, 197 samples were collected from 56 individuals. This includes 75 individuals aged 21 to 39 years enrolled from a workplace vaccination clinic and 122 individuals aged 64 to 95 years (51 enrolled from an aged-care living facility and 71 enrolled from a local health clinic). During the 2019/2020 study period, 147 samples were collected from 47 individuals, 42 from individuals aged 21 to 39 years enrolled from a workplace vaccination clinic, and 105 from individuals aged 64 to 95 years (41 enrolled from an aged-care living facility and 64 enrolled from a local health clinic) (summarized in Table 1 and detailed per study period in Table S1).

**TABLE 1:** Overall study participant demographics and PCR detection of *piaB* and *lytA* in saliva

Characteristics	Total		
	Workplace vaccination clinic	Aged-care living facility	Local health clinic
Total enrollment, n	34	30	39
Total no. of samples collected, n	117	92	135
Avg no. of samples per person, n (range)	3 (1 to 5)	3 (1 to 5)	3 (1 to 5)
Age in yr (median)	21 to 39 (28)	64 to 96 (88)	65 to 88 (72)
Female	23	16	21
*piaB*+, *lytA*+ samples, n (%)	14/117 (12.0%)	2/92 (2.2%)	13/135 (9.6%)
Period prevalence of pneumococcal carriage (*piaB*+ individuals), n (%)	7/34 (20.6%)	2/30 (6.7%)	10/39 (25.6%)
*piaB*−, *lytA*+ samples, n (%)	26/117 (22.2%)	2/92 (2.2%)	17/135 (12.6%)

### Detection of pneumococcus.

During the first study season (2018/2019) 16 of 197 (8.1%) culture-enriched saliva samples from 12 of 56 (21.4%) individuals tested qPCR-positive for both *piaB* and *lytA*, indicating the presence of pneumococcus. However, 27 (13.7%) samples from 14 (25.0%) individuals tested positive for *lytA* only. During the second study season (2019/2020) 14 of 147 (9.5%) culture-enriched saliva samples from 7 of 47 (14.9%) individuals tested qPCR positive for both *piaB* and *lytA*, and 18 (14.3%) samples from 9 (19.1%) individuals tested *lytA*-positive only. Several individuals were colonized with pneumococcus at multiple time points, including one individual who was colonized throughout the sampling period (Fig. 1). Pneumococcal colonization did not differ between the two study periods (odds ratio [OR], 1.25; 95% confidence interval [CI], 0.42 to 3.74), nor was colonization dependent on sex (OR, 1.24; 95% CI, 0.44 to 3.54). Individuals over 60 were less likely to be colonized with pneumococcus compared to younger study participants (63 to 80 year olds: OR, 0.71; 95% CI, 0.22 to 2.25; and >80 year olds: OR, 0.27; 95% CI, 0.42 to 3.74).

### Identification of nonpneumococcal Streptococcus species.

Through extensive reculture of the *lytA*-positive samples from the first season, we isolated 23 nonpneumococcal Streptococcus species strains (from 8 samples obtained from 5 individuals), which also tested qPCR-positive for only *lytA*. Colonization with *lytA*-positive nonpneumococcal Streptococcus species strains did not differ between the two study periods (OR, 1.08; 95% CI, 0.39 to 2.99). Individuals over 60 were also less likely to be colonized with *lytA*-positive nonpneumococcal Streptococcus species strains (63 to 80 year olds: OR, 0.38; 95% CI, 0.13 to 1.07; and >80 year olds: OR, 0.23; 95% CI, 0.04 to 1.36).

**FIG 2:**
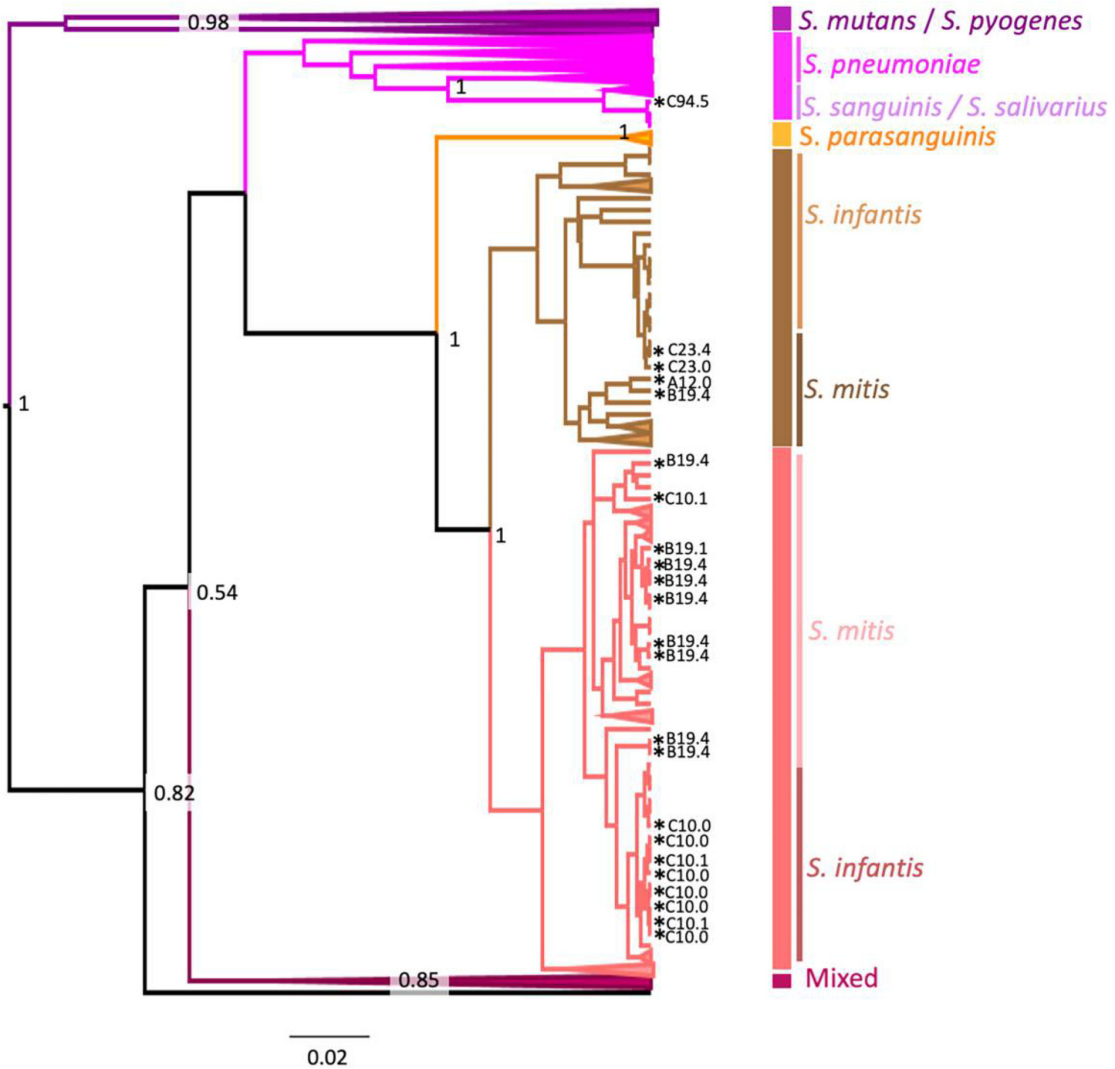
Bayesian maximum clade credibility (MCC) tree inferred using *rspB* gene sequences (431 nucleotides [nt]) derived from this study (*n* = 23) along with sequences representing the main Streptococcus spp. (*n* = 533). The mixed clade includes Streptococcus gordonii, Streptococcus equi, Streptococcus anginosus, Streptococcus constellatus, Streptococcus canis, and Streptococcus zooepidemicus. Clade credibilities of 50% and over are indicated in black at the relevant nodes. Clades are colored according to the main species found in the clade. Sequences from this study are marked with asterisks. Phylogenetic analysis was performed using the GTR + G4 + I nucleotide model. Samples are labeled with their study ID consisting of study site (A = workplace vaccination clinic, B = aged-care living facility, and C = local health clinic) and participant ID, followed by the time point at which the sample was collected (0 = day of influenza vaccination, 1 = 1 day after influenza vaccination, 4 = 28 days postvaccination, and 5 = 70 days postvaccination).

Of the 23 isolates that were *lytA*-positive and *piaB*-negative, 11 (48%) isolates were identified by S2 typing as Streptococcus mitis, 11 (48%) isolates were identified as Streptococcus infantis, and 1 (4%) isolate was identified as Streptococcus vestibularis (Table S2). The S. infantis isolates were in the same clade or a sister branch of the reference taxon for S. mitis, S. infantis, and Streptococcus parasanguinis (Fig. 2).

## DISCUSSION

The current gold standard method for the detection of pneumococcal carriage is testing nasopharyngeal swabs by conventional culture. Updated World Health Organization (WHO) recommendations made in 2013 by the Pneumococcal Carriage Working Group advised the inclusion of oropharyngeal swabs from adults when possible (20). A number of studies have demonstrated that the sensitivity of carriage detection can be further improved when molecular methods are applied (21–26) and when alternative sample types such as saliva are used (4, 12, 27). With increased use of molecular methods, there have been increased reports of confounding by nonpneumococcal Streptococcus spp., leading to uncertainty regarding the accuracy of culture-independent approaches (10, 13, 14, 22, 28). When implementing previously established molecular methods (12) to standardize approaches for the detection of pneumococcus in saliva from healthy adults between geographic locales, we noted potential confounding with a large proportion of saliva samples (45 of 103 (44%) individuals sampled) testing PCR positive for *lytA* but negative for *piaB*. This highlights the importance of testing for multiple specific gene targets when trying to accurately identify carriage of pneumococcus from complex oral samples.

Targeting the *lytA* gene has become widely accepted for the identification of pneumococcus. However, *lytA* homologues have been found in other Streptococcus spp. (29, 30), supporting the notion that detection of *lytA* alone can lead to pneumococcus misidentification (18). The gene target *piaB* is specific for encapsulated pneumococci but absent from other oral Streptococcus spp. (28, 31) and some certain unencapsulated pneumococcus strains (28, 32), meaning an underestimation of total pneumococcal carriage is possible. However, detection of both *lytA* and *piaB* genes by qPCR increases specificity and decreases false-positive detection for pneumococcus (15). Previous work has demonstrated concordance between *lytA* and *piaB* in the absence of unencapsulated or nonpneumococcal Streptococcus spp. with *lytA* homologues (11). Generally, when both *piaB* and *lytA* are detected in a sample at comparative Ct values, this is supportive of the presence of pneumococcus. However, it is not uncommon to observe stronger signals for *lytA*, indicating the copresence of a nonpneumococcus species also carrying the *lytA* gene.

In the current study, we found that the majority of the confounding signal in the *lytA* qPCR was caused by S. mitis and S. infantis. These results are in line with data from other studies detecting nonpneumococcal Streptococcus spp. from oral samples (13, 31, 32), further supporting that the WHO recommendation of using non-culture-based molecular methods for pneumococcal detection should be revised to include multiple targets (13, 31). Studies reporting on carriage rates detected from oral sample types when using only one qPCR target must be cautious regarding the specificity and interpretation of their results. Primers and probes are typically developed on pure isolates or evaluated *in silico* on genomic sequences available in public databases. However, the majority of available sequences are from strains isolated from cases of disease. With commensals rarely implicated in disease, the extent of gene homologues in nonpneumococcus Streptococcus commensals is largely unknown, although caution should be taken considering a high number of strains have been detected among the relatively small number of isolates tested in the current and other studies (28, 31). Interestingly, it was among the younger adults in this study population that were more likely to be colonized with these confounding Streptococcus spp. Thus, any assay developed for the detection of pneumococcal carriage, particularly in oral samples, should be thoroughly validated on both positive and negative samples to monitor potential of false positivity and how performance of the assay compares across different age groups. Accordingly, results from this study demonstrate the possibility of misidentification of Streptococcus spp. when solely utilizing the *lytA* qPCR assay to test oral samples, an observation that would not have been made if testing with *lytA* alone.

We acknowledge that sampling from 2020/2021 and beyond would be a significant addition to the data set presented here. However, the processing and analyzing of samples collected in carriage studies is an extensive process. As such, the data presented in the current study is within a time frame (sample collection to reporting) similar to the reports from surveillance on pneumococcal carriage conducted by others (4, 33, 34). Still, we feel that the data presented in this study remains relevant to the field as the issue of confounding of PCR assays used for the detection of pneumococcus was first reported much prior to this and still holds true today. Moreover, the carriage data presented here remain a valuable addition to the limited reports by others, providing insight into the period preceding the COVID-19 pandemic. Thus, these data can serve as a prepandemic baseline for both the confounding of molecular assays used for pneumococcal carriage detection and the rates of pneumococcal carriage. Ultimately, very few studies investigate these nonpneumococcal Streptococcus spp., despite their prevalence in carriage, yet it is important for those designing surveillance on pneumococcal carriage, especially as new vaccines are introduced, to be wary of this possibility of the risk for the false-positive detection of pneumococcus.

Understanding rates of carriage of pneumococcus in older adults is critical for evaluating vaccination strategies, both prior to and following their implementation. For this, nasopharyngeal swabbing has proved inefficient. Oral sample types improve carriage detection but need to be tested with care. Here, we detected a high frequency of samples testing qPCR-positive for *lytA* only, from which we isolated and identified nonpneumococcal species of Streptococcus responsible for this signal. This underscores the importance of testing for the presence of multiple gene targets in tandem for reliable molecular detection of pneumococcus in respiratory tract samples; targeting only *lytA* may lead to an overestimation of true carriage rates.

## MATERIALS AND METHODS

### Study design and population.

During the autumn/winter seasons (October to January) of 2018/2019 and 2019/2020, deidentified saliva samples were collected from healthy adults aged 21 to 39 years (community-dwelling) and ≥64 years (both community-dwelling and nursing home residents) as part of a study on influenza vaccination. Saliva was collected from study participants on the day of seasonal influenza vaccination (day 0) and on days 2, 7, 28, and 70. For the 2019/2020 study season, samples were not collected on day 2. Written informed consent was obtained from all participants, and the study was conducted in compliance with Good Clinical Practice and the Declaration of Helsinki of the World Medical Association. The study was approved by the Institutional Review Board of the Yale Human Research Protection Program (protocol ID 0409027018).

### Sample collection and processing.

All saliva samples were self-collected by study participants under supervision by trained study personnel, placed on wet ice, and transferred to the study laboratory at the Yale School of Public Health and processed within 4 h of collection. On arrival at the lab, 100 μL of each sample was cultured on blood agar plates supplemented with gentamicin (10%) (Remel, Lenexa, KS) (4). Following overnight incubation at 37°C with 5% CO_2_, all bacterial growth was harvested from culture plates into brain heart infusion (BHI) supplemented with 10% glycerol and stored at −80°C until further analysis. These culture-plate harvests were considered to be culture-enriched for pneumococci.

### Detection of pneumococcus.

Culture-enriched saliva samples were thawed on wet ice. DNA was extracted from 200 μL of each sample as previously described (19). Each DNA template was tested in qPCR for pneumococcal genes *piaB* (3, 11) and *lytA* (10). The assays were carried out in 20-μL reaction volumes using SsoAdvanced Universal Probe Supermix (Bio-Rad, USA), 2.5 μL of genomic DNA, and primer/probe mixes (Iowa Black quenchers) for *piaB* (1 μL) and *lytA* (1.2 μL) at concentrations of 200 nM. DNA of S. pneumoniae serotype 19F strain 64 (35) was included as a positive control in every run. The assays were run on a CFX96 Touch (Bio-Rad) under the following conditions: 95°C for 3 min, followed by 45 cycles of 98°C for 15 s and 60°C for 30 s. The samples were considered positive for pneumococcus when the *C_T_* values for both genes were ≤40 (36).

### Isolation of bacterial strains.

Culture-enriched saliva samples qPCR-positive for only *lytA* were revisited by culture in an attempt to isolate strains responsible for the *lytA* signal (11, 36). All strains isolated by reculture were retested in qPCR for *lytA* and *piaB* to confirm that the strain generating the *lytA*-positive and *piaB*-negative signal had been identified. Isolated strains were stored at −80°C until further processing.

### S2 typing.

The *lytA*-positive/*piaB*-negative strains were thawed on ice, cultured on blood agar plates using a 10-μL inoculating loop, and incubated overnight at 37°C with 5% CO_2_. Colonies were harvested using a 10-μL inoculating loop, and DNA was extracted via the boilate method (19). For each strain, the 408-bp variable region of the *rpsB* gene encoding the ribosomal protein S2 was amplified with conventional PCR using the primers previously described (28) with a modified protocol. Briefly, 2.5 μL of each DNA template was tested in 25-μL reaction volumes, consisting of 1 μL of each of the forward and reverse primers (10 μM each), 4 μL 5× Phusion HF buffer, 0.2 μL polymerase, 0.4 μL dNTP, and 10.4 μL DNase-free water. PCR conditions were as follows: 95°C for 15 min and then 40 cycles of 94°C for 30 s, 54°C for 1 min, and 72°C for 1 min, followed by 60°C for 30 min. DNA of S. pneumoniae serotype 19F strain 64 was included as a positive control in every run. Since amplicons generated with the S2 primers can vary in size (species dependent) (28), amplicons between 400 and 450 bp were gel purified using the QIAquick gel extraction kit (Qiagen). Each amplicon was adjusted to a final concentration of 60 ng, mixed with S2F (4 μM) or S2R (4 μM) primer, and sequenced by the Keck Biotechnology Resource Laboratory (Yale University, New Haven, CT, USA). Sequences generated were assembled using Geneious Prime 2022.1.1 (https://www.geneious.com) and cross-referenced with the reference data set of 501 streptococcal S2 sequences for species annotation. Sequences were aligned using Geneious Prime 2022.1.1, employing the Clustal Omega algorithm (37).

### Statistical analysis.

The study population was stratified into three age groups: younger adults (26 to 39 years old), older adults (60 to 79 years old), and the elderly (80 to 96 years old). The risk factors for pneumococcal carrier status were investigated by generalized estimation equations, and the strength of association was expressed as ORs with 95% CIs. Statistical analysis was performed using R version 3.6.1 with the following packages: dplyr (38), reshape2 (39), gee (40), and mgcv (41).
