# Supplementary material for: High Levels of Detection of Nonpneumococcal Species of Streptococcus in Saliva from Adults in the United States
Source: Microbiol Spectr. 2023 Apr 17;11(3):e05207-22. doi: 10.1128/spectrum.05207-22 (PMC10269540; doi:10.1128/spectrum.05207-22)
Supplement: Supplemental file 1 — Supplemental material. Download spectrum.05207-22-s0001.pdf, PDF file, 0.09 MB [file spectrum.05207-22-s0001.pdf]

SUPPLEMENTARY MATERIAL

High levels of detection of non-pneumococcal species of *Streptococcus* in saliva from adults in the USA

Maikel S. Hislop BS[1#], Orchid M. Allicock PhD[1#], Darani A. Thammavongsa BS[1], Sidiya Mbodj BS[1], Allison Nelson RN[2], Albert C. Shaw MD PhD[2], Daniel M. Weinberger PhD[1*], Anne L. Wyllie PhD[1*]

[1]Department of Epidemiology of Microbial Diseases, Yale School of Public Health, New Haven, Connecticut, USA; [2]Department of Internal Medicine, Section of Infectious Diseases, Yale School of Medicine, New Haven, Connecticut, USA.

[#]Contributed equally
*Co-senior authors

Corresponding author:
Anne Wyllie PhD
anne.wyllie@yale.edu
Yale School of Public Health, LEPH 823, 60 College St, New Haven, CT 06510

Table S1. Study participant demographics and PCR detection of *piaB* and *lytA* genes in saliva collected from adults visiting (A) a workplace vaccination clinic, (B) an aged-care living facility, or (C) a local health clinic, by study period and overall.

**Study year**	**2018/2019**			**2019/2020**			**Total**		
Study site	A	B	C	A	B	C	A	B	C
Total enrollment, n	20	16	20	14	14	19	34	30	39
Total number of samples collected, n	75	51	71	42	41	64	117	92	135
Average number of samples per person, (range)	4 (1-5)	3 (1-5)	4 (1-5)	3 (1-4)	3 (1-4)	3 (1-4)	3 (1-5)	3 (1-5)	3 (1-5)
Age in years (median)	21-39 (29)	64-95 (89)	65-88 (73)	23-35 (27)	66-96 (82)	65-85 (71)	21-39 (28)	64-96 (88)	65-88 (72)
Female	13	8	11	10	8	10	23	16	21
piaB+/lytA+ samples, n (%)	8/75 (10.7%)	1/51 (2.0%)	7/71 (9.9%)	6/42 (14.3%)	1/64 (1.6%)	7/64 (10.9%)	14/117 (12.0%)	2/92 (2.2%)	13/135 (9.6%)
Period prevalence of pneumococcal carriage (*piaB*+ individuals), n (%)	5/20 (25.0%)	1/16 (6.3%)	6/20 (30.0%)	2/14 (14.3%)	1/14 (7.1%)	4/19 (21.0%)	7/34 (20.6%)	2/30 (6.7%)	10/39 (25.6%)
piaB-/lytA+ samples, n (%)	14/75 (18.7%)	2/51 (3.9%)	11/71 (15.5%)	12/42 (28.6%)	0/64	6/64 (9.4%)	26/117 (22.2%)	2/92 (2.2%)	17/135 (12.6%)

Table S2. Identification of non-pneumococcal *Streptococcus* spp. generating positive signal in the *lytA*-PCR assay widely-used for pneumococcus detection, when testing saliva samples collected from adults visiting (A) a workplace vaccination clinic (21-39 year olds), (B) an aged-care living facility (≥64 year olds), and (C) a local health clinic (≥64 year olds).

**Setting**	**Study ID**	**Timepoint (day)**	**Isolate ID**	**Species identified by S2-typing (tree alignment)**	**NCBI blast of *rpsB* sequence generated by S2-typing**
A	12	0	5	*S. infantis*	*S. oralis/oral/VT*
B	19	2	2	*S. mitis*	*S.mitis*
B	19	70	4	*S. mitis*	*S. mitis*
B	19	70	5	*S. mitis*	*S. mitis/S. pseudopneumoniae*
B	19	70	6	*S. mitis*	*S. mitis*
B	19	70	8	*S. mitis*	*S. mitis/S. pseudopneumoniae*
B	19	70	1.1	*S. mitis*	*S. mitis/S. oralis*
B	19	70	4.1	*S. mitis*	*S. mitis/S. sp oral taxon*
B	19	70	5.1	*S. mitis*	*S. mitis/S. pseudopneumoniae*
B	19	70	7.1	*S. mitis*	*S. mitis/S. pseudopneumoniae*
B	19	70	8.1	*S. mitis*	*S. mitis/S. pseudopneumoniae*
C	10	0	2	*S. infantis*	*S. sp oral taxon/S. mitis*
C	10	0	3	*S. infantis*	*S. sp oral taxon/S. mitis*
C	10	0	8	*S. infantis*	*S. sp oral taxon/S. mitis*
C	10	0	2.1	*S. infantis*	*S. sp. oral taxon/S. mitis*
C	10	0	3.1	*S. infantis*	*S. sp. oral taxon/S. mitis*
C	10	0	8.1	*S. infantis*	*S. sp. oral taxon/S. mitis*
C	10	2	4	*S. mitis*	*S. mitis/S. pseudopneumoniae*
C	10	2	4.1	*S. infantis*	*S. sp. oral taxon/S. mitis*
C	10	2	5.1	*S. infantis*	*S. mitis/S. pseudopneumoniae*
C	23	0	1.2	*S. infantis*	*S. oralis/S. sp. oral taxon*
C	23	70	7.1	*S. infantis*	*S. oralis/S. sp. oral taxon*
C	94	0	5	*S. sanguinis*	*S. vestibularis/S. thermophilus/S. salivarius*
